# Fissure of the Soft and Hard Palate; Operation

**Published:** 1852-10

**Authors:** 


### ARTICLE XX.

### *Fissure of the Soft and Hard Palate; Operation.* By Mr. Avery.

We dilated a little time ago upon the important improvements which have been introduced in the operation for cleft palate by Mr. Fergusson, and we are glad to find that this new method of operating is more and more adopted by practical surgeons. Mr. Avery, amongst others, has published the very satisfactory results which he has obtained from the division of certain muscles connected with the soft palate, and as we had a few days ago an opportunity of seeing Mr. Avery operate at this hospital, upon a very complicated case, we shall just point out the facts which most attracted our attention:

The patient (a young woman about twenty-four years of age) was born with harelip, and a fissure both in the hard and soft palate. The harelip was formerly rectified in the country, but the cleft palate had not been interfered with. The most interesting portion of the operation was the difficult task of detaching the tough tissues adherent to the hard palate, and lined with mucous membrane. Mr. Avery used for that purpose two instruments, which rendered him great service: a scalpel, the cutting portion of which is at right angles with the stem, and scissors whose extremities are disposed in the same manner. These instruments, when introduced between the soft parts and the bone, will cut laterally; but it is nevertheless very difficult to render the former flaccid by disconnecting them sufficiently from the bone, as the locality offers a great many obstacles. Nothing of the kind could, however, be attempted, were not the surgeon aided by the courage and perseverance of the patient; the latter displayed in this instance great patience and fortitude.

The hemorrhage was somewhat considerable, but Mr. Avery at last succeeded in detaching the soft parts from the bone, to the extent of half an inch transversely; and so disconnected were they, that they assumed all round the fissure a whitish aspect, and were quite flaccid. One point worthy of notice is, that by the above-mentioned measures the irritability of the air-passages and fauces becomes so blunted, that the subsequent passing of the ligatures produces hardly any cough or uneasiness. Mr. Avery, after having pared the margin of the soft fissure, placed the first of his threads, by the ordinary manipulation, about an inch behind the incisor teeth, and the four others (the threads being of alternate thickness) at short distances from one another down to the uvula.

It was now seen that the two halves of the soft palate did not meet so readily as the division of the levator palati and palato-pharyngeus might have led the surgeon to expect, but the apposition became quite perfect after Mr. Avery had freely carried his knife transversely through the anterior arch of the soft palate (palato-glossus.) A small aperture is left close to the incisor teeth, which, by means of the blistering fluid, will, in all probability, unite, though it should be recollected that the ligatures over the hard palate are very apt to tear through the structures.

We have merely given our impressions, in some degree, after witnessing this operation, and shall be happy to note down the final results of the case. It is by no means unlikely that great strides will, before long, be made in the mode of proceeding for the rectification of fissure of the hard palate, if we may judge from what we have seen this day.—*London Lancet.*

